# The impact of experiential avoidance on anxiety and depressive disorders in hematological cancer patients

**DOI:** 10.1007/s10865-025-00553-2

**Published:** 2025-02-09

**Authors:** Gregor Weißflog, Jochen Ernst, Peter Esser, Uwe Platzbecker, Vladan Vucinic, Anja Mehnert-Theuerkauf, Franziska Springer

**Affiliations:** 1 Department of Medical Psychology and Medical Sociology, Comprehensive Cancer Center Central Germany (CCCG), University Medical Center Leipzig, Leipzig, Germany; 2 Family Counseling Center, Volkssolidarität Stadtverband Leipzig e.V., Betriebsstätte Wurzen, Germany; 3 Medical Clinic and Policlinic 1, Hematology and Cellular Therapy, Comprehensive Cancer Center Central Germany (CCCG), University Hospital Leipzig, Leipzig, Germany; 4 University Hospital Carl Gustav Carus, Dresden, Germany; 5 Department of Medical Psychology and Medical Sociology, University Medical Center Leipzig, Philipp-Rosenthal-Straße 55, 04103 Leipzig, Germany

**Keywords:** Anxiety disorders, Depressive disorders, Cancer, Hematologic malignancies, Experiential avoidance, Transdiagnostic factor

## Abstract

Anxiety disorders and/or depressive disorders co-occurring with hematological cancer are an additional burden for patients. Experiential avoidance (EA; efforts to avoid negative emotions, thoughts, or memories) is an empirically evident transdiagnostic factor for the onset and maintenance of anxiety and depressive disorders in non-cancer populations. There is lack of evidence on the impact of EA in predicting anxiety and depression in cancer patients. A total of 291 patients with hematological cancer (60% male, mean age 55 years) were included in this cross-sectional observational study. Participants were assessed using the Structured Clinical Interview for DSM-5 mental disorders (SCID-5). EA was assessed via self-report using the Brief Experiential Avoidance Questionnaire (BEAQ). Hierarchical binomial logistic regression was conducted in order to estimate the impact of EA on anxiety and depressive disorders. A total of 38 patients (13.3%) met the diagnostic criteria for a current anxiety disorder, while 49 patients (17.2%) met the criteria for a current depressive disorder. In bivariate analyses, EA was significantly elevated in patients with an anxiety disorder in comparison to those without (54.4 vs. 48.9; *p* = 0.01). The same was true for depressive disorder (54.9 vs. 48.6; *p* < 0.01). After controlling for relevant sociodemographic and medical factors, EA did not predict anxiety or depressive disorder in separate regression models. The presence of an anxiety disorder was significantly predicted by female sex, younger age and elevated comorbidity burden. In contrast, the presence of a depressive disorder was predicted by comorbidity burden. Sociodemographic and medical predictors have greater predictive potential than EA regarding current anxiety and depressive disorder in hematological cancer patients.

**Supplementary Information:**

The online version contains supplementary material available at 10.1007/s10865-025-00553-2.

## Introduction

Hematological cancer patients are vulnerable to a range of health risks associated with both their illness condition as well as the often-invasive treatments they undergo. The somatic impact is significant, especially regarding the cancer- and treatment related fatigue or potential treatment complications like Graft-versus-Host-Disease (*GvHD*). The psychological impact is also considerable with patients experiencing declining quality of life and elevated distress levels as well as elevated levels of uncertainty, anxiety and depression (Allart-Vorelli et al., 2015; Amonoo et al., 2019; Hansen et al., 2023; Smith, 2015; Stark & House, 2000).

The results of a meta-analysis indicate that the prevalence of anxiety disorders in hematological cancer patients is 10.3% according to the DSM (including versions III, III-R and IV) based on data from six original studies. The prevalence of depression was 18.3% in the USA and slightly lower with 11.6% in the UK (Mitchell et al., 2011). Data from a large epidemiological study from Germany report a 4-week prevalence of 12.9% of any anxiety disorder and 6.3% of any mood disorder in hematological cancer patients (Mehnert et al., 2014).

A whole range of factors influence the occurrence of comorbid anxiety and depressive disorders in patients with cancer. Sociodemographic characteristics, such as younger age, female sex, and lack of partnership or social support, are associated with a higher prevalence of anxiety and depression (Goerling et al., 2024; Niedzwiedz et al., 2019). A second group of predictors relates to medical characteristics, including disease-related factors, such as type of cancer (highest for breast, head and neck cancer), advanced stage, comorbidity and some types of treatment as factors increasing the risk for comorbid mental disorders (Goerling et al., 2024; Götze et al., 2018; Niedzwiedz et al., 2019; Smith, 2015). A third set of predictors includes psychological variables. A pre-existing mental disorder before cancer or the use of psychological services were identified as predictive factors for elevated rates of anxiety disorders or depression in cancer patients (Niedzwiedz et al., 2019). Personality variables such as neuroticism also contribute to a higher prevalence of comorbid mental disorders (Harrison & Maguire, 1994; Niedzwiedz et al., 2019).

Experiential Avoidance (EA), a component of the framework of acceptance and commitment therapy (ACT), predicts the onset, relapse and maintenance of anxiety disorders and depression in non-cancer populations (Grogans et al., 2023; Spinhoven et al., 2014, 2017). Per definition, EA is a psychological phenomenon that occurs when a person: (1) is unwilling to remain in contact with aversive experiences, including emotions, bodily sensations, thoughts and memories, and (2) takes action to alter these events or experiences which includes different forms of avoidance (Hayes et al., 1996). EA can manifest itself as cognitive avoidance, e.g. suppressing thoughts, as emotional avoidance with denial or blunting, and as behavioral avoidance with distraction and distancing. In the context of cancer, EA may involve avoiding thoughts about treatment or treatment side effects, reassurance seeking, disengaging from social life or avoiding emotions related to uncertainty about the future. A recent review of EA in (advanced) cancer patients revealed both advantages and disadvantages of EA, with it appearing beneficial in the short term by alleviating distress, but dysfunctional in the longer term by impairing adequate coping or a value-driven and self-efficacious life as a cancer survivor (Davis et al., 2023). The authors of the review conclude that previous empirical results of studies investigating EA in the context of cancer are inconclusive and further research is needed. To our knowledge, there is only one single study investigating EA in hematological cancer patients, especially those with a hematopoietic stem cell transplantation (HSCT). In this longitudinal study with 111 patients, EA had no predictive value in predicting recovery of psychological and physical functioning following HSCT (Larson et al., 2019).

The contribution of EA as a psychological predictor, and its interaction with other predictors, regarding the presence of anxiety and depressive disorders in hematological cancer patients remains unclear. Therefore, our study aims to address this gap in knowledge by answering the following research questions: (1) To what extent do hematological cancer patients report EA in comparison with BEAQ validation samples? (2) Is EA associated with the presence of current anxiety and depressive disorders within the context of further potential sociodemographic and medical predictors?

## Methods

### Design and participants

In this cross-sectional observational study, cancer patients were included if they had been (i) diagnosed with any hematological malignancy, myelodysplastic syndrome or other neoplasms of uncertain or unknown behavior of lymphoid, hematopoietic and related tissue (ICD-10: C81-C96, D46, D47), (ii) were aged between 18 and 70 years, (iii) were fluent in German, (iv) were able to provide informed consent, (v) had been or were currently undergoing active cancer treatment and (vi) had no plans of re-admission at the time of study inclusion.

Patients were consecutively recruited between April 2019 and September 2021 at different wards and the outpatient clinic of the Clinic for Hematology, Cellular Therapy and Hemostaseology at University Medical Center Leipzig. In a first recruitment wave, eligible patients were recruited by their treating physicians and were asked for their consent to be contacted by the study team. Interested patients were then directly contacted by a study member and were informed about the study. Additionally, some patients joined the study after it had been presented at two patient networks. Due to the COVID-19 pandemic and to reduce direct contact and an additional burden on physicians, we had to adapt our recruitment procedure. In a second recruitment wave, a study member screened all patients scheduled for the outpatient clinic via review of the medical charts. Eligible patients were contacted directly by the study team via phone or mail and were informed about the study. Patients who refused to participate were asked for their reason for non-participation. The primary research aim of the study was the assessment of the prevalence of trauma- and stressor-related disorders, post-traumatic stress disorder (PTSD) and adjustment disorder (AD) according to updated diagnostic criteria (Springer et al., 2023). The study protocol was approved by the ethics committee of the Medical Faculty at the University of Leipzig (447/17-ek) and has been published (Esser et al., 2019). All patients provided written informed consent prior to study participation.

### Data collection

Assessment took place a minimum of six weeks after the end of cancer treatment (e.g. stem cell transplantation) to ensure that no additional burden was placed on the patients by the study assessment and that acute treatment-related stressors were no longer present. However, participants receiving permanent cancer treatment were directly assessed with the interview and questionnaire after providing informed consent, meaning their assessment took place during their active treatment. In contrast, patients with acute treatment burdens, such as stem cell transplantation, were assessed a minimum of six weeks after completing their treatment. The assessment was conducted with a structured clinical interview for DSM-5 in person or via phone and a paper-pencil questionnaire with validated tools to be filled in at home. Patients were reminded via phone if the questionnaire was missing every 2–4 weeks and up to five times. The timing of reminders was adjusted to the respective clinical status of the patient, e.g. hospitalization or feeling of burden. After complete study assessment, i.e. interview and questionnaire, participants received 20 Euro as an incentive.

### Measures

Sociodemographic and medical data was extracted from the medical charts, i.e. age, sex, diagnosis, time since diagnosis, treatment with SCT and remission status. Additional information was assessed via self-report within the questionnaire, e.g. comorbidity burden as the number of comorbidities with impairments ≥ 4 on a scale from 0 = not at all to 5 = very much (Bayliss et al., 2005).

### Clinical interviews

With the Structured Clinical Interview for DSM-5 - Clinical Version (SCID-5) the following diagnoses were assessed: current depressive disorders including major/ recurrent depression and current persistent depressive episode with respected times frames according to DSM-V and current anxiety disorders including generalized anxiety disorder, agoraphobia, specific phobia, and panic disorder. SCID training was initially conducted by an experienced psychotherapist in conducting SCID interviews and was afterwards standardized for additional interviewers who joined later. Standardization of interview training encompassed introduction of the SCID-5 including a case study, work shadowing during at least two interviews and being supervised in the first two interviews. Individual differential diagnostic questions were discussed within the study team including the experienced psychotherapist.

### Questionnaires

#### BEAQ

Experiential avoidance was assessed with the *Brief Experiential Avoidance Questionnaire* (BEAQ), a short form of the *Multidimensional Experiential Avoidance Questionnaire* (Gamez et al., 2011, 2014), which is validated in German language (Schaeuffele et al., 2022). The 15 items are rated on a 6-point Likert scale from 1 (“strongly disagree”) to 6 (“strongly agree”). Higher values indicate higher experiential avoidance. The total scale enables calculation of a sum score from 15 to 80. The instrument shows good internal reliability (α = 0.77–0.80) and acceptable validity in different validation samples (Gamez et al., 2014; Schaeuffele et al., 2022). According to (Gamez et al., 2014), the results of four subscales can be presented (for subscale and item scores from this study see supplement 1). In our study sample, reliability was good for the total BEAQ scale (Cronbach Alpha = 0.85), good for the subscale ‘*explicit avoidance behavior*’ (Cronbach Alpha = 0.84) and acceptable for ‘*attitudes/beliefs regarding distress*’ (Cronbach Alpha = 0.70). The reliability for ‘*implicit avoidance*’ was borderline acceptable (Cronbach Alpha = 0.66). The reliability for ´*ability to respond effectively to distress´* was not calculated because the subscale consists of only one item. The total score of hematological cancer patients was compared with two German BEAQ validation samples (Schaeuffele et al., 2022). The first sample consists of a student population (*n* = 596; mean = 44.77, standard deviation = 10.19) and the second sample comprises a combination of outpatient and online therapy patients (*n* = 182; mean = 51.77, standard deviation = 10.60).

### Statistical analysis

Descriptive statistics were presented for the entire sample for sociodemographic and medical data, as well as data on EA (total scale, subscale and items). The Shapiro-Wilk-Test was used to test for normal distribution of data. The effect size Cohen´s d was calculated to estimate the clinical relevance of mean differences between different samples. In preparation of the regression analyses, the associations of sociodemographic and medical variables as well as EA with the dichotomous outcomes anxiety disorder and depressive disorder were estimated using Spearman and point-biserial correlations. Additionally, t-tests or chi square tests were calculated when predictors and/or outcomes were dichotomous. Hierarchical binomial logistic regressions were calculated for current anxiety or depressive disorder in order to estimate the contribution of particular blocks of predictors to explaining variance (Nagelkerkes R^2^). A multistep approach was used, incorporating relevant predictors from an inter-correlation matrix. The variables sex and age form block 1 (sociodemographic predictors). Medical variables of stem cell transplantation (sct) yes/no, remission status yes/no, and comorbidity burden represent block 2 (medical predictors). The variable experiential avoidance total score was entered as block 3. Multicollinearity of the included predictors was tested with the indices variance inflation factor (< 5) and conditional index (largest value < 30). Hosmer-Lemeshow-Test was used to check the model fit of the regression model with non-significant results indicating good model fit. All analyses were conducted with SPSS 29^®^.

## Results

### Sample characteristics

**Fig. 1:**
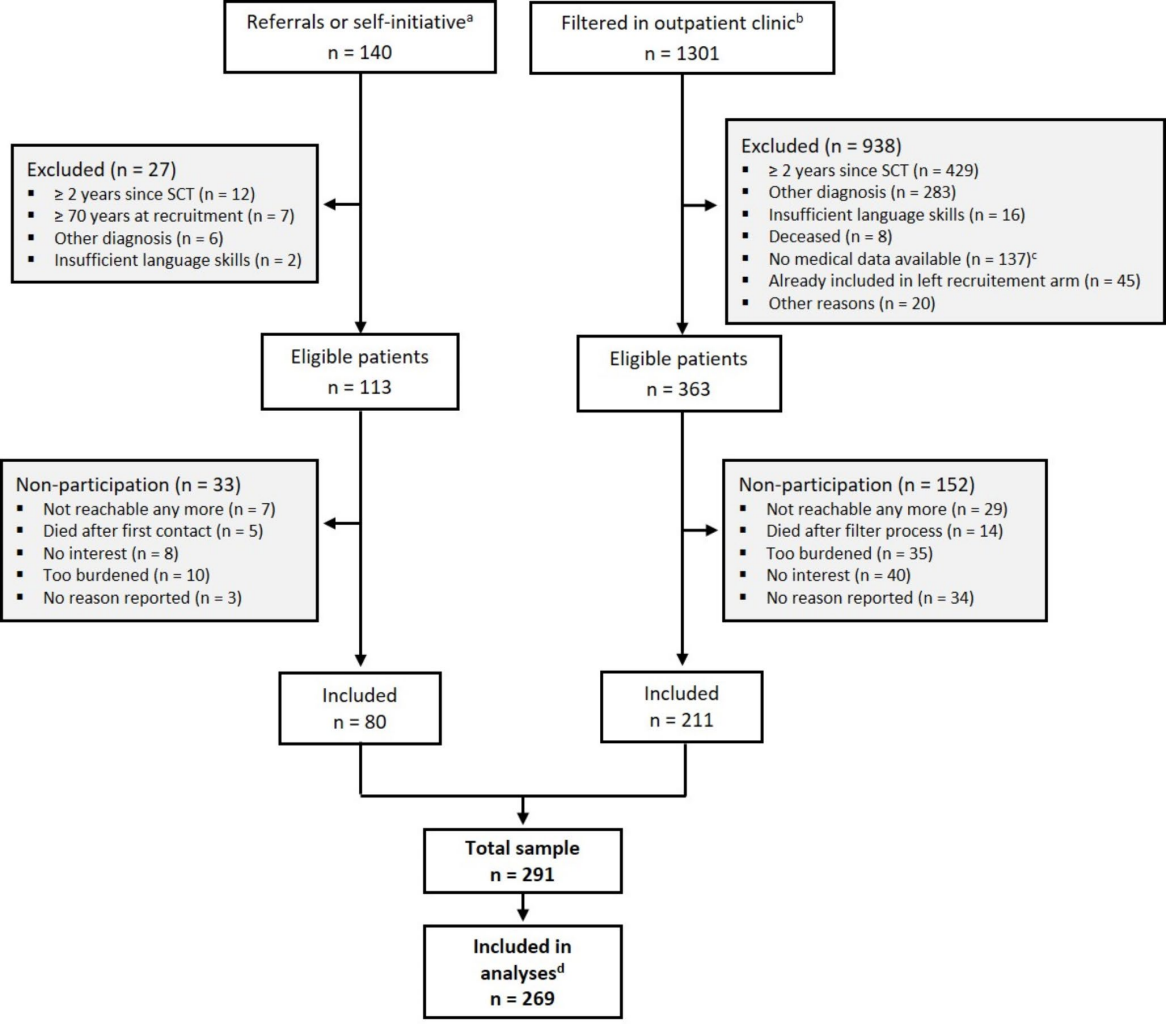
Flow-Diagram. SCT, stem cell transplantation. ^a^ referred by physicians at the study center or practices and self-initiated contact of interested patients; ^b^ all patients between 18 and 70 years who were scheduled for the outpatient clinic at the days of recruitment; ^c^ no medical information in the records, e.g. stem cell donors or patients from other institutions seeking second medical opinion; ^d^ participants with SCID-5 and BEAQ data

In total, 291 hematological cancer patients were included in this study, with 269 patients completing the SCID-5 and BEAQ (see Fig. 1). Median time interval between interview and questionnaire is one week and 75% of patients lay between 0 and 2.3 weeks. 171 patients (60%) were male, and the mean age was 55 years (Table 1). The most common cancer diagnoses were Myeloid myeloma (ICD-10 code: C90, *n* = 67, 23%) and Myeloid leukemia (acute and chronic; ICD-10 code: C92, *n* = 67, 23%) accounting for almost half of the total sample.

**Table 1:** Sample characteristics

	Total sample (*n* = 291)
Age; mean (SD, range)	54.5 (12.5, 19–71)
Gender	
Female	117 (40.2)
Male	174 (59.8)
Education	
≤ 10 years	131 (48.3)
> 10 years	140 (51.7)
Employment	
Currently working	119 (40.9)
Not working because of cancer	83 (28.5)
Retired	69 (23.7)
Unemployed	12 (4.1)
Diagnosis	
Hodgkin Lymphoma	19 (6.5)
Non-follicular Lymphoma	38 (13.1)
Multiple Myeloma	67 (23.0)
Lymphoid Leukemia	29 (10.0)
Myeloid Leukemia	67 (23.0)
Myelodysplastic Syndrome	26 (8.9)
Other	45 (15.5)
Time since diagnosis in months; mean (SD), median	24.3 (33.9), 13
Remitted	101 (40.6)
Relapse	32 (11.7)
Treatment	
Chemotherapy	231 (79.4)
Radiation	56 (19.2)
Antibody	56 (19.2)
Surgery	33 (11.3)
Allogeneic SCT	81 (27.8)
Autologous SCT	83 (28.5)
Other Treatment	47 (16.2)

*Note* Displayed n and %, if not otherwise noted; most variables were self-reported in the questionnaire, thus percentages are based on valid answers in the questionnaire and sometimes not the full sample; Stem cell transplantation (SCT), standard deviation (SD)

In total, 38 patients (13.3%, *n* = 26 female) had a current anxiety disorder. 22 patients (7.7%, *n* = 14 female) were diagnosed with GAD, 12 patients (4.2%, *n* = 9 female) with specific phobia, 6 patients (2.1%, *n* = 5 female) with panic disorder, and two patients (0.7%, *n* = 1 male) with agoraphobia. Forty-nine patients (17.2%, *n* = 25 male) had a current depression (including major depression: *n* = 26, 9.1%, recurrent depression: *n* = 19, 6.7%, and persistent depressive disorder: *n* = 19, 6.7% (including *n* = 15 with “double depression”)). Thirty-nine persons had one diagnosis, 23 persons had two diagnoses and four persons had three diagnoses.

### Experiential avoidance

The mean total score of BEAQ (Min, Max: 15, 80) was 49.65 (SD = 11.86). The means of all BEAQ subscale scores are presented in Table 2. The mean total score of the BEAQ was normally distributed, the BEAQ subscales scores were not. The total BEAQ score and all subscales were significantly associated with depressive disorders yielding correlations between *r* = 0.14 and *r* = 0.28. The mean total score of the BEAQ and further two subscales were significantly associated with anxiety disorders yielding correlations of *r* = 0.16 (total BEAQ score), *r* = 0.21 (implicit avoidance) and *r* = 0.20 (ability to respond effectively to distress). The comparisons with two samples of a German validation study yielded a difference of Cohens d = 0.46 in comparison with the student sample and indicates a small effect. In comparison with the psychotherapy patient sample, Cohens d was 0.19, indicating no effect.

**Table 2:** BEAQ inter-correlation matrix and bivariate associations with outcomes

				Depressive disorders		Anxiety disorders	
	Mean	Min, Max	SD	*r*	*p*	*r*	*p*
BEAQ total	49.65	15, 80	11.86	**0.19**	**< 0.01**	**0.16**	**0.01**
explicit avoidance behavior	28.22	8, 48	7.36	**0.14**	**0.02**	0.11	0.08
attitudes/beliefs regarding distress	13.64	4, 24	4.41	**0.16**	**0.01**	0.09	0.13
implicit avoidance	5.27	2, 12	2.33	**0.28**	**< 0.001**	**0.21**	**< 0.001**
ability to respond effectively to distress	2.83	1, 6	1.38	**0.18**	**< 0.01**	**0.20**	**< 0.01**

### Impact of sociodemographic, medical factors and experiential avoidance on anxiety and depressive disorders

The bivariate associations of the included predictors and outcomes are shown in Table 3. There are significant results for the associations with both mental disorders of comorbidity burden and EA, and additionally for the variable sex with current anxiety disorder.

**Table 3:** Association of mental disorders and experiential avoidance (EA), sociodemographic and medical factors

	Anxiety disorder			Depressive disorder		
	Yes (*n* = 38)	No (*n* = 247)	*p* (t-test)	Yes (*n* = 49)	No (*n* = 236)	*p* (t-test)
Age (years)	51.1	55.1	0.07	54.7	54.5	0.94
No. of comorbidity burden with ≥ 4	2.53	1.10	< 0.001	2.63	1.01	< 0.001
EA (sum score)	54.4	48.9	0.01	54.9	48.6	< 0.01
	n (%)	n (%)	*p* (χ^2^)	n (%)	n (%)	*p* (χ^2^)
Sex						
Female	26 (23)	88 (77)		24 (21)	90 (79)	
Male	12 (7)	159 (93)	< 0.001	25 (15)	146 (85)	0.16
SCT						
Yes	17 (11)	141 (89)		27 (17)	131 (83)	
No	21 (17)	106 (83)	0.15	22 (17)	105 (83)	0.96
Remission^1^						
Yes	8 (8)	93 (92)		11 (11)	90 (89)	
No	24 (16)	125 (84)	0.06	29 (19)	120 (81)	0.07

*Note* EA Experiential avoidance, SCT stem cell transplantation

^1^*n* = 35 with no data for variable remission

### Anxiety disorder

No multicollinearity was observed for any of the regression models presented below. The binomial logistic regression model for the outcome anxiety disorder was significant (χ²(6) = 26.88, *p* < 0.001, R^2^ = 0.21) with a good model fit (χ²(8) = 5.93, *p* = 0.66). Significant predictors for the presence of a current anxiety disorder were *sex*, *age* and *comorbidity burden* (see Table 4). EA was not significant when controlled for sociodemographic and medical factors (*p* = 0.14).

**Table 4:** Associated factors with occurrence of anxiety disorder and depressive disorder

Outcome Predictor	Anxiety Disorder			Depressive disorder		
***+ Sociodemographic characteristics***						
*** Model statistics***	***χ*** ^***2***^	***p***	***R*** ^***2***^	***χ*** ^***2***^	***p***	***R*** ^***2***^
	11.19	< 0.01	0.09	1.66	0.44	0.01
	***Exp (B)***	***p***		***Exp (B)***	***p***	
Sex	0.33	0.01		0.65	0.28	
Age	0.95	< 0.01		0.98	0.21	
***+ Medical characteristics***						
*** Model statistics***	***χ*** ^***2***^	***p***	***R*** ^***2***^	***χ*** ^***2***^	***p***	***R*** ^***2***^
	24.64	< 0.001	0.19	28.47	< 0.001	0.20
	***Exp (B)***	*p*		***Exp (B)***	***p***	
SCT (yes, no)	1.24	0.64		1.23	0.62	
Rem (yes, no)	0.53	0.22		0.63	0.33	
Comorb	1.37	0.01		1.60	< 0.001	
***+ Experiential Avoidance***						
** Model statistics**	***χ*** ^***2***^	***p***	***R*** ^*2*^	***χ*** ^***2***^	***p***	***R*** ^***2***^
	26.88	< 0.001	0.21	29.82	< 0.001	0.21
	**Exp (B)**	**p**		**Exp (B)**	**p**	
Exp. Avoid.	1.03	0.14		1.02	0.25	

*Note* predictor parameters (Exp (B), p, R^2^) within this table are for the full model (including all three blocks), model statistics (*χ²*, p, R^2^) are presented blockwise

### Depressive disorder

The binomial logistic regression model for the outcome depressive disorder was statistically significant (χ²(6) = 29.82, *p* < 0.001, R^2^ = 0.21) with a good model fit (χ²(8) = 10.35, *p* = 0.24). The only significant predictor for the presence of a current depressive disorder was *comorbidity burden* (see Table 4). EA was not significant when controlled for sociodemographic and medical factors (*p* = 0.25).

## Discussion

### Interpretation

The first research question of this study was to identify levels of EA within hematological cancer patients. Therefore, the extent of EA in hematological cancer patients was compared with two German comparison samples (Schaeuffele et al., 2022). These two comparisons led to the extent of EA in our sample being classified as increased compared to the younger general population and as equal in relation to a patient sample undergoing psychological treatment (outpatient or online psychotherapy). The comparison with the healthy sample from the general population shows that EA is elevated in hematological cancer patients indicating that EA is a psychologically relevant mechanism for dealing with mental burden, also in patients undergoing hematological cancer treatment.

The second research question was to examine if EA is able to explain the presence of current anxiety and depressive disorders in hematological cancer patients. In bivariate analyses, the total score of EA was significantly associated with depressive disorder (*r* = 0.19; *p* < 0.01) and with anxiety disorder (*r* = 0.16; *p* = 0.01). Despite showing a significant association of EA and anxiety or depressive disorders in hematological cancer patients, the bivariate correlation is rather weak, representing limited clinical relevance (*r* < 0.20).

On a subscale level, most subscale associations with the outcomes anxiety and depressive disorder were not exceeding total scale associations. There are two exceptions: the subscales *implicit avoidance* and *ability to respond effectively to distress*. *Implicit avoidance* yielded a significant association (and higher than the total scale) with depressive disorder (*r* = 0.28) and anxiety disorder (*r* = 0.21). This subscale is characterized by a feeling of being disconnected from emotions. *Ability to respond effectively to distress* also yielded significant associations with both disorders, but only for anxiety disorder exceeding total scale association (*r* = 0.20). The wording of this single item subscale is *“Fear/anxiety won’t stop me from doing important things”*, which therefore directly targets the mental representations of coping with burdensome experiences. This suggests that in particular the cognitive-emotional dimension of EA is associated with depressive and anxiety disorders in this sample of hematological cancer patients. For depressive disorders, there were significant associations for the two further subscales, but with general weak correlations (*r* = 0.14 to *r* = 0.18). For anxiety disorders, associations with the two further subscales were non-significant (*r* = 0.09 to *r* = 0.11).Summarizing these findings, it can be helpful to support hematological cancer patients by “giving a name” to negative feelings which are associated with the cancer and its treatment. This could be a starting point for an enhanced coping, e.g. through the processes of cognitive change (Webb et al., 2012) or acceptance including mindfulness (Sauer et al., 2024). This is in line with the already mentioned study of Larson et al., which found that hematological cancer patients with a better ability to describe internal experiences and to act with awareness reported less anxiety and depression (Larson et al., 2019). Here, acceptance and commitment therapy (ACT) could provide a suitable therapeutic framework to prevent patients from developing habitual cognitive and behavioral avoidance strategies within the cancer context. From the lens of functional analysis, EA is dysfunctional when it leads to cycles of negative reinforcement and inflexible avoidance-based thinking and behavior, while at the same time limiting values-guided actions. Using the therapeutic processes of the hexaflex model of ACT like mindfulness, acceptance and cognitive fusion, patients can learn to better distinguish between the helpful aspects of avoidance that are beneficial in the short term (e.g. ignoring a pain experience and meet a friend) and those that are dysfunctional in the long term (e.g. the inflexible pattern of withdrawal from social life associated with unwanted, recurrent pain experiences).

In contrast to the above-mentioned significant bivariate associations, EA revealed no significant contribution beyond sociodemographic (age, sex) and medical predictors (in this study above all comorbidity burden) in multivariate regression models. The total variance explanations of the regression models for anxiety disorders and for depressive disorders (both with R^2^ = 0.21) are good, indicating an appropriate selection of predicting factors. The sociodemographic factors younger age and female sex were associated with the presence of an anxiety disorder. This is consistent with previous research findings (Götze et al., 2020; Niedzwiedz et al., 2019). The finding that comorbidity burden has a strong predictive value for anxiety and depressive disorders adds to the previous finding that comorbidity burden is associated with lower quality of life in cancer patients (Götze et al., 2020).

In combination with these described sociodemographic and medical factors, no additional contribution of EA in predicting anxiety or depressive disorders can be demonstrated. A possible explanation for this finding could be that avoidance in cancer patients is sometimes difficult, as experiences and emotions come up repeatedly due to ongoing and often invasive cancer treatment. Although patients with explicit and implicit avoidance of cognitions and emotions have an increased risk of developing a depressive and anxiety disorder as shown for the bivariate associations, the additional comorbidity burden has a greater impact on the presence of depressive or anxiety disorders in hematological cancer patients. This suggests that comorbidity management in hematological cancer patients should be of particular importance, including for the prevention of depressive and anxiety disorders.

### Limitations

Although using a large sample and conducting thorough assessment including clinical interviews and validated diagnoses, this study has several limitations. The cross-sectional design of our study did not allow us to draw conclusion about the impact of EA on the development of anxiety and depressive disorder over time. Further, the role played by premorbid (pre-cancer) mental disorders and the stability of EA over the life course cannot be answered with sufficient reliability based on the available data. Not all disorders of the anxiety spectrum were included, such as social phobia and obsessive- compulsive disorder (limiting the term “anxiety disorders”). In addition, some constructs of interest, such as psychosocial support, neuroticism, rumination, or intolerance of uncertainty, that might further explain experiences of EA in cancer patients were not assessed and future studies may investigate these constructs (Panjwani et al., 2024; Spinhoven et al., 2017). Measures of neurobiological dysfunctions representing biotypes of anxiety and depressive disorders were not included in this study, although this would be an interesting research avenue (Tozzi et al., 2024).

## Electronic supplementary material

Below is the link to the electronic supplementary material.

Supplementary Material 1

## Data Availability

Deidentified data may be available upon reasonable request to the authors.
