# Supplementary material for: The impact of experiential avoidance on anxiety and depressive disorders in hematological cancer patients
Source: J Behav Med. 2025 Feb 9;48(2):394–402. doi: 10.1007/s10865-025-00553-2 (PMC11929714; doi:10.1007/s10865-025-00553-2)
Supplement: Supplementary file 1 — Supplementary Material 1 [file 10865_2025_553_MOESM1_ESM.docx]

Supplement 1.

| **BEAQ** | **MW** | **SD** |
| --- | --- | --- |
|  |  |  |
| **Total score** | **49.65** | **11.86** |
|  |  |  |
| ***Subscale ´explicit avoidance behavior´*** |  |  |
| 02. I´m quick to leave situations that make me uneasy | 3.84 | 1.23 |
| 03. I try to put unpleasant memories out of mind | 3.77 | 1.30 |
| 05. I won’t do something unless I absolutely have to | 2.95 | 1.37 |
| 08. I rarely do things that might upset me | 3.48 | 1.37 |
| 10. I try to put off unpleasant tasks for as long as possible | 3.27 | 1.40 |
| 11. I go out of my way to avoid uncomfortable situations | 3.53 | 1.34 |
| 13. I work hard to keep out upsetting feelings | 3.88 | 1.43 |
| 14. I won’t do something if I have doubts | 3.53 | 1.38 |
|  |  |  |
| ***Subscale ´attitudes/beliefs regarding distress´*** |  |  |
| 01. The key to a good life is never feeling pain | 3.01 | 1.58 |
| 07. I would give up a lot not to feel bad | 3.72 | 1.49 |
| 12. A big goal is to be free from painful emotions | 3.66 | 1.52 |
| 15. Pain always leads to suffering | 3.32 | 1.55 |
|  |  |  |
| ***Subscale ´implicit avoidance´*** |  |  |
| 04. I feel disconnected from my emotions | 2.56 | 1.32 |
| 09. It´s hard for me to know what I am feeling | 2.72 | 1.39 |
|  |  |  |
| ***Subscale ´ability to respond effectively to distress´*** |  |  |
| 06. Fear/anxiety won’t stop me from doing important things | 2.83 | 1.38 |

Note. BEAQ = Brief Experiential Avoidance Questionnaire. The 15 items are rated on a 6-point Likert scale from 1 (“strongly disagree”) to 6 (“strongly agree”). Item 6 is reversely coded. Higher values indicate higher experiential avoidance. Range of the total score between 15 and 80.
